# The emergence of generative artificial intelligence platforms in 2023, journal metrics, appreciation to reviewers and volunteers, and obituary

**DOI:** 10.3352/jeehp.2024.21.9

**Published:** 2024-04-11

**Authors:** Sun Huh

**Affiliations:** Institute of Medical Education, Hallym University College of Medicine, Chuncheon, Korea; Hallym University, Korea

## The emergence of generative artificial intelligence platforms in 2023 and the effect of the COVID-19 pandemic on clinical performance

After ChatGPT’s release on November 30, 2022, generative artificial intelligence (AI) fever was a hot topic in 2023. Many kinds of generative AI platforms appeared, including GPT-4, Bing, Gemini (former Bard), Claude.ai, Clova X, and Wrtn. The issues related to ChatGPT adoption discussed in research articles mainly dealt with passing tests, applicability in medical practice, and writing support [1].

Many manuscripts on generative AI have also been submitted to this journal. Most of them were accepted if the methods and interpretations were sound. Out of them, my brief report on ChatGPT’s performance on a parasitology exam, with a 60.8% correct answer rate [2], was the first article to be published in the journal on the performance of ChatGPT. Another remarkable article was written by a team of 1st year medical students. As a class assignment, they wrote an article comparing the performance of 6 generative AI platforms by information amount, accuracy, and relevance [3]. Their writing was quite impressive, with a core message focusing on the usefulness of generative AI platforms. The conclusion was also very informative—“A Korea-based company’s generative AI, Clova X, showed 100% relevance to the queries in Korea, which is the best performance out of the 6 generative AI platforms. The experience of using generative AI in the classroom enhanced the authors’ self-efficacy, which led to a heightened interest in the subject matter.” Dr. Ju Yoen Lee, a copyright law professor, wrote an article on AI authorship [4], which has also been a hot topic regarding the use of generative AI. She concluded, “Current AI chatbots such as ChatGPT are much more advanced than search engines in that they produce original text, but they still remain at the level of a search engine in that they cannot take responsibility for their writing. For this reason, they also cannot be authors from the perspective of research ethics.”

The journal’s editorial policies on the use of generative AI in article writing and peer review have been announced [5]. The main difference from other journal publishers is that the *Journal of Educational Evaluation for Health Professions* (JEEHP) does not ask authors to disclose the use of AI tools. The reason for this is that the editorial office is not able to screen the use of AI tools consistently, although multiple similarity check tools are used.

The fever of using generative AI in medical or health professions education will continue in 2024. The effects of using generative AI platforms will be a new topic in educational evaluation for health professions.

In Taiwan, coronavirus disease 2019 (COVID-19) negatively impacted medical students’ clinical performance, regardless of their specialty [6]. This paper provides evidence regarding the challenges met during the COVID-19 pandemic, even though the pandemic led to the more active use of online tools and virtual reality environments in education.

## Journal metrics and statistics

The first 2022 Journal Impact Factor (JIF, 4.4) arrived in 2023 [7]. The 2022 Journal Citation Indicator was 0.96, and 0.58 in 2021. The 2023 JIF may be announced this June. It is expected to be more than 9.2. The CiteScore 2023 by Elsevier was 9.5 on April 5, 2024. These high journal metrics values originated from some review articles on sample size estimation [8], educational applications of the metaverse [9], and e-learning during the COVID-19 pandemic [10]. We hope to achieve a more even citation frequency for all articles, although doing so is challenging.

**Fig. 1.**
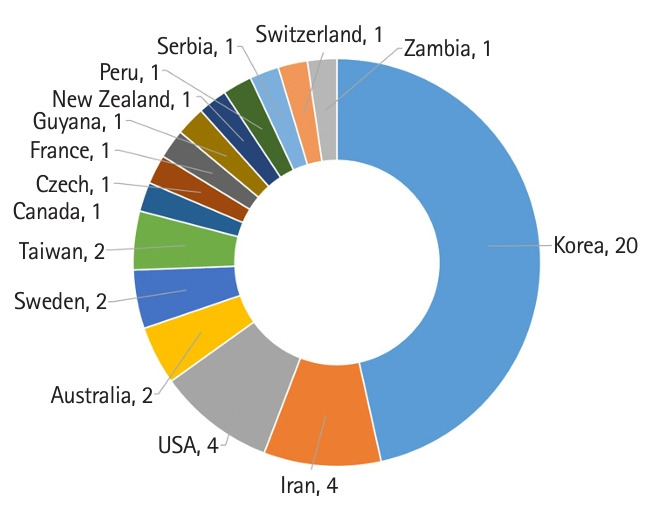
Number of articles in *Journal of Educational Evaluation for Health Professions* according to the authors’ country in 2023.

Fig. 1 shows the authors’ countries in the 2023 issue. Journal statistics for 2023 are presented in Table 1.

The acceptance rate of peer-reviewed manuscripts, including commissioned and unsolicited manuscripts, was 80.6% (38/47). However, if only unsolicited manuscripts are counted, the acceptance rate was 77.5% (31/40), somewhat exceeding the rate from 2022 (70.8%) [11]. The editorial office will do its best to use reviewers’ time effectively. Fortunately, the median time to the first decision was shortened from 20 days in 2022 to 15 days in 2023. The journal’s goal is 14 days.

## Appreciation to reviewers and volunteers

In 2023, 67 reviewers were invited from 17 countries, as follows. Their dedication makes it possible to maintain the journal’s quality:

**Australia:** Boaz Shulruf, The University of New South Wales

**France:** Guillaume Decormeille, University of Toulouse

**Indonesia:** Daniel Ardian Soeselo, Atma Jaya Catholic University of Indonesia; Pandji Winata Nurikhwan, Lambung Mangkurat University

**Iran:** Sara Adarvishi, Ahvaz Jundishapur University of Medical Sciences; Masoumeh Albooghobeish, Ahvaz Jundishapur University of Medical Sciences; Samane Ghasemi, Isfahan University of Medical Sciences; Abdolreza Gilavand Ahvaz, Jundishapur University of Medical Sciences; Rizevandi Parisa Kermanshah, University of Medical Sciences; Nooshin Sarvi Sarmeydani, Ahvaz Jundishapur University of Medical Sciences; Abdolghani Abdollahimohammad, Zabol University of Medical Sciences

**Israel:** Jacob Urkin, Ben-Gurion University

**Italy:** Stefania Chiappinotto, Università di Udine

**Korea:** Sukhee Ahn, Chungnam National University; Eunbeen Bae, Korea Health Personnel Licensing Examination Institute; Su Jin Chae, University of Ulsan; Ara Cho, The Catholic Unversity of Korea; Year Hur, Hallym University; Geum Hee Jeong, Hallym University; Eunbo Kang, Dong-Eui Institute of Technology University; Yunsoo Kim, Catholic Kwandong University; Young-Min Kim, The Catholic Unversity of Korea; Sun-Hee Kim, Daegu Catholic University; Seon-Kyoung Kim, Dong-Eui University; Mi Young Kim, Hallym University; Ji-Eun Kim, Hallym University; Dong Joon Kim, Hallym University; Hyun Kyoung Kim, Kongju National University; Kyung Won Kim, Seoul Women’s University; Han Joe Kim, Yonsei University; Hyun Young Koo, Daegu Catholic University; Ju Yoen Lee, Hanyang University; Ji Young Lim, Inha University; Cheonghwan Lim, Hanseo University; Seung-JooNa, CHA University; Younjae Oh, Hallym University; Song Yi Park, Dong-A University; Sun Nam Park, Seoul Women’s College of Nursing; Janghee Park, Soonchunhyang University; Seong HoPark, University of Ulsan; Dong Gi Seo, Hallym University; Yun Joo Seo, InfoLumi; Youlhun Seoung, Cheongju University; Sanghee Yeo, Kyungpook National University; Nayoung Yi, Daejeon University; Jeong-Ju Yoo, Soonchunhyang University

**Morocco:** Aziz Naciri, Hassan II University of Casablanca

**New Zealand:** Marcus Henning, University of Auckland

**Norway:** Anna Pasetto, University of Oslo

**Peru:** Grace Huertas, Universidad Cientifica del Sur

**Serbia:** Dragan Bogdanović, State University of Novi Pazar; Natasa Milic, University of Belgrade; Vedrana Pavlovic, University of Belgrade

**Sweden:** Jonas Söderholm, Karolinska Institutet; Xiaoshan Zhou, Karolinska Institutet

**Taiwan:** Chung-Hsien Chaou, Chang-Gung Memorial Hospital; Pin-Hsiang Huang, National Yang-Ming University

**Thailand:** Chanokporn Anu-un, Sunpasitthiprasong Hospital; Siwat Bhumiwat, HRH Princess Chulabhorn College of Medical Science; Krittaphas Kangwanrattanakul, Burapha University; Tipaporn Kanjanarach, Khon Kaen University

**Turkey:** Hanife Durgun, Ordu University; Berna Köktürk Dalcali, Bandırma Onyedi Eylül University; Nuray Turan, Istanbul University-Cerrahpasa

**United States:** Derrick Ferguson, Campbell University of St. Augustine for Health Sciences; Gail Jensen, Creighton University; Hwanggyu Lim, Graduate Management Admission Council

Tom Huh, a graduate student in the Division of Life Sciences, College of Life Sciences and Biotechnology, Korea University, Seoul, Korea, recorded some audio abstracts voluntarily.

I apologize to submitters whose manuscripts were not included in the peer review process or rejected after peer review. This is inevitable due to space (budget) limitations originating from the lack of an article processing charge. Although the editorial team and peer reviewers can work voluntarily, payments to publishing companies cannot be reduced to maintain top-tier quality of the style and format and the journal homepage. To overcome this limitation, the editorial office will continue to discuss the author-side payment of the article processing charge with the publisher.

## Obituary

**Fig. 2.**
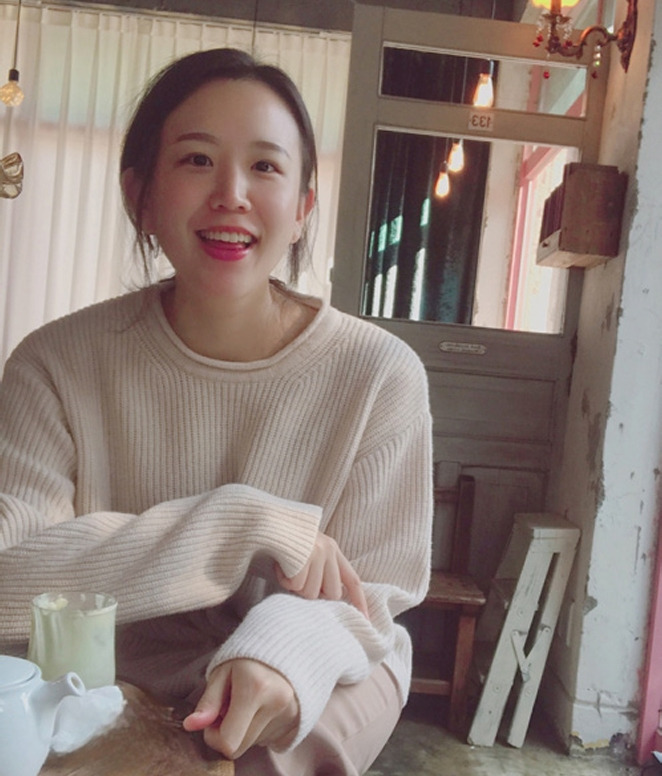
Photo of the late Ms. Jinyoung Cho (The photo was provided by her family).

Ms. Jinyoung Cho (1989–2024), who had worked as an editorial assistant since 2023, passed away on March 27, 2024, due to postpartum amniotic fluid embolism (Fig. 2). This embolism is a rare puerperium complication, from which the number of deaths in Korea was 6, 9, and 10 in 2018, 2019, and 2020, respectively. The number of deaths per 100,0000 population was 1.8, 3.0, and 3.7 in 2018, 2019, and 2020 [12].

There is no way to express our grief over her death. She was passionate in her work for the journal and sincerely desired its further development. Her contributions played an invaluable role in helping JEEHP achieve its current status as an international top-tier journal in its field. The editorial management team will always remember her dedication to the journal. She left behind her husband and daughter. I hope her daughter grows up to become a leader who will continue her mother’s legacy and contribute to our society.

## Figures and Tables

**Table 1.** Journal statistics of manuscripts submitted to the *Journal of Educational Evaluation for Health Professions* from January 1 to December 31, 2023

	No.	Content
Manuscripts submitted	268	
No. of commissioned manuscripts	7	Editorials, 4; reviews, 3
No. of unsolicited manuscripts	261	
Manuscripts rejected without peer review	221	Unsuitable, 206; other reasons, 11; rejected before review, 4
Manuscripts peer-reviewed out of 268 submitted manuscripts	47	Published, 38 (80.6%); rejected, 9
No. of publications out of 268 submitted manuscripts	38	
Acceptance rate overall (%)	14.2	38/268=0.142
Acceptance rate of unsolicited manuscripts (%)	11.9	31/261=0.119
Median time from submission to the first decision (days)	15	
Median time from submission to publication (days)	37	
Median time from acceptance to publication (days)	1	
